# Generation of Codon-Optimized Fad3 Gene Transgenic Bovine That Produce More n-3 Polyunsaturated Fatty Acids

**DOI:** 10.3390/ani15010093

**Published:** 2025-01-03

**Authors:** Guanghua Su, Zhuying Wei, Chunling Bai, Danyi Li, Xiaoyu Zhao, Xuefei Liu, Lishuang Song, Li Zhang, Guangpeng Li, Lei Yang

**Affiliations:** 1 State Key Laboratory of Reproductive Regulation and Breeding of Grassland Livestock (R2BGL), Inner Mongolia University, 24 Zhaojun Rd., Hohhot 010070, China; weizhuying2008@126.com (Z.W.); chunling1980_0@163.com (C.B.); lidanyi0215@163.com (D.L.); zhaoxiaoyu3233@163.com (X.Z.); liuxuefei1006@126.com (X.L.); xiaoshuang2000@126.com (L.S.); zhanglinmg@aliyun.com (L.Z.); gpengli@imu.edu.cn (G.L.); 2 College of Life Sciences, Inner Mongolia University, 24 Zhaojun Rd., Hohhot 010070, China

**Keywords:** n-3 PUFAs, fatty acid desaturase 3 (Fad3), transgenic cattle

## Abstract

Polyunsaturated fatty acids (PUFAs) such as linoleic acid (18:2, n-6) and α-linolenic acid (18:3, n-3) are essential for the growth, development, and well-being of mammals. However, most mammals, including humans, cannot synthesize n-3 and n-6 PUFAs and these must be obtained through diet. The beneficial effect of converting n-6 polyunsaturated fatty acids (n-6 PUFAs) into n-3 polyunsaturated fatty acids (n-3 PUFAs) has led to extensive research on the flax fatty acid desaturase 3 (*Fad3*) gene, which encodes fatty acid desaturase. Still, the plant-derived *Fad3* gene is used much less in transgenic animals than the Fat-1 gene from Caenorhabditis elegans. To address this problem, we used somatic cell nuclear transfer (SCNT) technology to create codon-optimized Fad3 transgenic cattle. Gas chromatographic analysis showed that the n-3 PUFA content of transgenic cattle increased significantly, and the ratio of n-6 PUFAs to n-3 PUFAs decreased from 3.484 ± 0.46 to about 2.78 ± 0.14 (*p* < 0.05). In conclusion, *Fad3* gene knock-in cattle are expected to improve the nutritional value of beef and can be used as an animal model to study the therapeutic effects of n-3 PUFAs in various diseases.

## Simple Summary

This study successfully created fatty acid desaturase 3 (*Fad3*) transgenic cattle by utilizing CRISPR-Cas9 technology to insert a codon-optimized *Fad3* gene sequence into bovine fibroblast cells and employing somatic cell nuclear transfer (SCNT) technology. Gas chromatographic analysis confirmed that the n-3 PUFA (polyunsaturated fatty acid) content in the transgenic cattle was significantly increased, while the ratio of n-6 PUFAs to n-3 PUFAs decreased. Fad3 transgenic cattle are rich in polyunsaturated fatty acids and represent a high-quality breed of beef cattle. The successful breeding of Fad3 transgenic cattle not only meets the demand for healthy diets but also serves as a model for studying the effects of endogenous n-3 PUFAs on animals.

## 1. Introduction

Polyunsaturated fatty acids (PUFAs) contain more than two double bonds and are essential for the health of mammals, including humans [1]. PUFAs are primarily categorized into two groups: n-6 PUFAs and n-3 PUFAs. n-6 PUFAs are mainly found in plants, terrestrial animals, and aquatic products, where the first unsaturated bond is between the sixth and seventh carbon atoms from the terminal methyl group. This group includes linoleic acid, γ-linolenic acid, and arachidonic acid [2,3]. n-3 PUFAs are mainly found in plants and deep-sea fish [4], and are characterized by the first unsaturated bond located between the third and fourth carbon atoms from the terminal methyl group. This group mainly includes α-linolenic acid, EPA (eicosapentaenoic acid), and DHA (docosahexaenoic acid) [5]. Functionally, n-3 PUFAs are crucial components of cell membranes. They also act as substrates for certain enzymes and signaling molecules, and as regulatory factors affecting gene expression [6]. Additionally, n-3 PUFAs play a significant role in lipid synthesis, lipid deposition, adipocyte maturation, differentiation, apoptosis, and lipid oxidative metabolism [7]. Epidemiological studies have shown that the intake of n-6 PUFAs in people’s daily diets is often excessive, while the intake of n-3 PUFAs is insufficient [8]. Excessive intake of n-6 PUFAs is strongly associated with inflammation and other diseases [9]. Therefore, the balance of n-6/n-3 PUFAs is of great biological significance and closely related to human health [8,9].

The fatty acid desaturase 3 (*Fad3*) gene is derived from flax and is primarily responsible for the production of PUFAs in plants. It encodes the ∆15 position fatty acid desaturase, which converts n-6 PUFAs to n-3 PUFAs, thereby increasing n-3 PUFA levels and reducing the n-6/n-3 PUFA ratio [10]. To date, animal studies have focused more on the *Fat-1* gene, which functions similarly to *Fad3*. The Fat-1 gene is derived from *Caenorhabditis elegans* and encodes the n-3 polyunsaturated fatty acid desaturase. *Fat-1* transgenic animals have increased n-3 PUFA levels [11,12,13]. Research on *Fat-1* transgenic mice have shown that this gene has a positive role in anticancer effects [14,15], anti-inflammatory effects [16,17], oxidative damage reduction [17,18], and lipid metabolism [19,20,21]. These transgenic mice can serve as effective models for further study. Subsequently, the Fat-1 gene was applied to livestock, resulting in the creation of Fat-1 transgenic pigs and transgenic dairy cows. In transgenic pigs, the n-6/n-3 PUFA ratio in the main tissues was significantly decreased [12,13], while the n-3 PUFA content in the somatic cells and milk of transgenic cows was significantly higher than that of normal cows [22,23]. This advancement opens up new avenues for humans to obtain polyunsaturated fatty acids from a broader range of sources.

Building on previous research, we conducted a similar study on the plant-derived *Fad3* gene in mice [24]. Given the positive expression of the *Fad3* gene in mice, we utilized the Fad3 gene to construct an overexpression vector for cell transfection. Transgenic cattle were produced by using somatic cell nuclear transfer technology and the contents of polyunsaturated fatty acids were analyzed by gas chromatography. This study aims to produce bovine animals carrying a codon-optimized *Fad3* gene, measuring their capacity to synthesize polyunsaturated fatty acids and validating the expression efficiency of the *Fad3* gene in livestock. These results are intended to offer crucial references and assistance for the breeding of cattle.

## 2. Materials and Methods

### 2.1. Ethical Statement

All of the animal procedures and experiments were carried out in accordance with the guidelines of the Council of China Animal Welfare and were approved by the Institutional Animal Care and Use Committee at Inner Mongolia University (approval ID: IMU-CATTLE-2017-045).

### 2.2. Cell Primary Culture

Fibroblasts isolated from fetal skin of Luxi Yellow Cattle were employed as primary cells for the production of transgenic cattle. Fetuses, obtained by surgical extraction at 45 days of gestation, were minced in a 100 mm Petri dish and subsequently soaked in 75% ethanol for 10 s, followed by three consecutive washes in PBS containing 2% Pen Strep (Gibco, New York, NY, USA) for 20 s each. The fetal skin tissue was separated, with the tissue pieces then soaked in fibroblast culture medium (DMEM F12, Gibco, New York, NY, USA, supplemented with 10% FBS, Gibco, New York, NY, USA and 1% Pen Strep) for 10 s. The tissue was spread flat in a 100 mm dish, inverted, and incubated at 5% CO_2_ and 38.5 °C. After 4 h, the dish was retrieved, 7–8 mL of culture medium was added, and the dish was returned to the incubator for continued culture.

### 2.3. Cell Transfection

To perform cell transfection, 1 µg of Cas9 plasmid (a combination of 0.25 µg each of Cas9 vectors for 4851, 4358, 4844, and 4347 sgRNAs) was mixed with 0.5 µg of the Fad3 gene expression cassette fragment. The plasmid mixture was then combined with 30 μL of opti-MEM and mixed gently. PLUS reagent (2 μL diluted in opti-MEM, Gibco, New York, NY, USA to 20 μL) was added to the plasmid mixture, mixed well, and allowed to stand at room temperature for 5 min. LTX reagent was prepared by diluting 3.5 μL of LTX in opti-MEM to reach 50 μL, mixed gently, and then added to the DNA-PLUS mixture, mixed gently, and allowed to stand at room temperature for 30 min (15338-100, Thermo, New York, NY, USA). For cell transfection, 100 μL of opti-MEM was added to the adherent cells, and the DNA-LTX mixture was gently added dropwise, mixed slightly, and incubated at 37 °C for 6 h before being replaced with standard culture medium. The transfection results were observed 48 h post-transfection.

### 2.4. DNA Extraction

Genomic DNA was extracted using the TIANamp Genomic DNA Kit (W9104, TIANGEN, Hilden, Germany). The cells were collected in a 1.5 mL EP tube, and 20 μL of proteinase K was added and mixed well. Then, 200 μL of buffer GB was added, fully inverted and mixed well, and incubated at 70 °C for 10 min. After that, 200 μL of absolute ethanol was added, fully shaken and mixed well for 15 s, and briefly centrifuged to remove any water droplets from the inner wall of the tube cap. Next, 500 μL of buffer GD was added to the adsorption column CB3 and centrifuged at 12,000 rpm for 30 s, the waste liquid was discarded, and the adsorption column CB3 was placed into a new collection tube. Then, 600 μL of rinsing solution PW was added to the adsorption column CB3 and centrifuged at 12,000 rpm for 30 s, the waste liquid was discarded, and the adsorption column CB3 was placed into the collection tube again. This step was repeated, after which the adsorption column CB3 was placed into the collection tube and centrifuged at 12,000 rpm for 2 min, the waste liquid was discarded, and it was allowed to dry. The adsorption column CB3 was then placed into a clean collection tube, 50 μL of elution buffer was added in suspension, left at room temperature for 2–5 min, and centrifuged at 12,000 rpm for 2 min, and the eluate was collected in a centrifuge tube. Finally, the sample was stored at −20 °C.

### 2.5. RNA Extraction and Reversal

The cells were collected in a 1.5 mL EP tube, placed in an ice box, and mixed well with 300 μL of RNA-plus. After thorough mixing, an additional 700 μL of RNA-plus was added and shaken evenly. The centrifuge was set to 4 °C and the sample was centrifuged at 12,000 rpm for 5 min. The supernatant was transferred to a new 1.5 mL EP tube, and then 200 μL of chloroform was added and shaken evenly, left to stand for 5 min at 4 °C, and centrifuged at 12,000 rpm for 5 min. The supernatant was then transferred to a new 1.5 mL EP tube, an equal volume of isopropanol was added, and the mixture was inverted and mixed well. After standing at room temperature for 10 min, the supernatant was discarded, and the precipitate was retained. Next, 1 mL of absolute ethanol was added, inverted, and mixed well, followed by centrifugation at 12,000 rpm at 4 °C for 5 min. The supernatant was discarded, the precipitate was dried at room temperature, and 30 μL of nuclease-free water was added. The sample was stored at −80 °C. The extracted RNA was then used as a template and reverse-transcribed into cDNA according to the Takara reverse transcription reagent manual. (RR047A, Takara, Beijing, China).

### 2.6. Preparation of Fad3 Transgenic Cattle

The bovine SCNT protocols were the same as in our previous reports [25]. Briefly, bovine ovaries were collected from a local slaughterhouse and kept in physiological saline solution containing penicillin and streptomycin at 20–25 °C. Cumulus–oocyte complexes (COCs) were collected from 3 to 8 mm diameter antral follicles using needles. The COCs were then cultured in four-well plates (Nunc) in 500 µL of oocyte maturation media (medium-199 containing 10% FBS, 20-ng/mL EGF (Sigma, Livonia, MI, USA), 1-µg/mL 17 β-estradiol (Gibco, New York, NY, USA), 1-µg/mL FSH (Sigma, USA), and 0.1-IU/mL LH (Sigma, USA)) at 38.5 °C under 5% CO_2_. After 24 h of maturation, the oocytes were transferred by pipette into 1 mg/mL hyaluronidase to remove the cumulus cells. The oocytes with the first polar bodies were used as recipient oocytes for nuclear transfer.

The positive transgenic cells were micro-manipulated and placed into the perivitelline space of enucleated oocytes. The successfully reconstructed embryos underwent electrofusion (Cryologic, Victoria, Australia) 30 min later and were subsequently cultured in SOF medium at 38.5 °C under conditions of 5% CO_2_ saturation and high humidity for 30 min. All of the fused embryos were activated in 5 µmol/L ionomycin for 5 min, followed by a 5 h incubation period in 10 µg/mL cycloheximide. The embryos were then transferred to SOF developmental medium, covered with mineral oil, and cultured at 38.5 °C under 5% CO_2_ saturation and high humidity. After 48 h, the cloned embryos were co-cultured with a granulosa cell layer up to blastocyst stage. Then, the cloned transgenic embryos were transferred into the selected recipient surrogate cows by using synchronous estrus treatment. Afterwards, pregnancy tests were conducted at 60 days, 120 days, and 210 days of pregnancy to calculate the pregnancy rate.

### 2.7. PUFA Analysis

The PUFA analysis was performed as previously described [26]. Briefly, Fad3 transgenic bovine cells and wild-type cells were digested with 0.05% trypsin, counted, and washed twice with PBS before being centrifuged at 1500 rpm for 5 min. After discarding the supernatant, 1 mL of 2.5% H_2_SO_4_/methanol solution was added, and the samples were incubated in a water bath at 80 °C for 90 min. Once cooled to room temperature, 1.5 mL of 0.9% NaCl solution and 1 mL of n-hexane were added, shaken for 5 min, and centrifuged at 1500 rpm for 5 min to extract the fatty acids. The organic phase was transferred to a new tube, mixed with a saturated KOH–methanol solution, shaken for 5 min, and centrifuged at 1500 rpm for 10 min. The upper layer was collected in a GC-MS sample vial for fatty acid analysis. The gas chromatographic conditions included helium as the carrier gas, an HP-88 column, a constant linear velocity of 20.0 cm/s, a separation ratio of 20.0%, and a 1 μL injection volume. The temperature program started at 60 °C for 1 min, increased to 140 °C at 40 °C/min, was held for 10 min, then increased to 240 °C at 4 °C/min, and was held for 15 min.

### 2.8. Statistical Analysis

Statistical analysis was carried out using GraphPad Prism 9.0 software. The fatty acid content in each group of cells and tissues was expressed as mean ± SEM. One-way ANOVA was used for comparing the means of Fad3 transgenic and non-transgenic cattle samples. Statistical significance was determined using the Holm–Sidak method, with alpha = 0.05. Each row was analyzed individually, without assuming a consistent SD. * *p* < 0.05 was considered to have statistical significance, with ** *p* < 0.01 and *** *p* < 0.001 indicating extremely significant differences.

## 3. Results

### 3.1. Humanization of the Fad3 Gene

**Figure 1:**
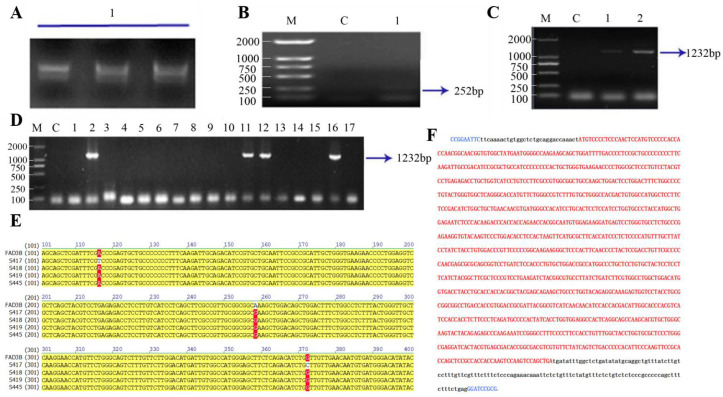
Humanization of the *Fad3* gene. (**A**) The electrophoresis result of total RNA in flax seeds. (**B**) RT-PCR detection of the internal reference gene Act1, M: DL2000, C: H_2_O. (**C**) Cloning the *Fad3B* gene, M: DL2000, C: H_2_O. (**D**) Detecting positive colonies, M: DL2000, C: H2O. 1–17: Randomly picking 17 cloned colonies. (**E**) Sequencing of the *Fad3B* gene. (**F**) The sequence of the codon-optimized Fad3. Red regions represent the coding sequence (CDS) areas of genes.

Total RNA was extracted from flax seeds widely cultivated in north-western China. Electrophoresis showed 28S and 18S subunit bands, with the 5S subunit band relatively indistinct (Figure 1A). After reverse transcription, a 252 bp band was amplified using the internal reference gene Act1 (Actin-related gene 1) primer (Figure 1B). The *Fad3* gene was then amplified using flax cDNA as the template (Figure 1C). The obtained fragments were ligated to T vectors, and 17 colonies were picked for bacterial liquid PCR detection after transformation (Figure 1D). Four positive clonal colonies were selected for sequencing, and the sequencing results were labeled S417, S418, S419, and S445. Comparing the *Fad3* gene sequence on GenBank with the sequencing results, it was found that only one site in the sequenced sample S418 was different from the *Fad3b* gene sequence on GenBank: a mutation site (A→G) at base 225. In all four sequenced samples, the base is G (Figure 1E) and codes for valine (Ala), with no change in the amino acid sequence.

Since the *Fad3* gene sequence is derived from plants, it is necessary to optimize the Fad3 gene sequence (see Supplementary Material S1 for details). As humans are the ultimate beneficiaries, the codons were optimized according to the human codon preference table, ensuring all codons match the optimal codons of *Homo sapiens*. The gene was then further modified based on the abundance of specific codon tRNAs and the low abundance of rare codon tRNAs in human cells. Considering the impact of DNA methylation on gene expression, the gene was re-modified according to the CG-enriched regions in its upstream regulatory region. Additionally, synonymous codon substitution was performed in regions with high CG enrichment within the gene. RNA Structure software (RNAfold web server URL is http://rna.tbi.univie.ac.at/cgi-bin/RNAWebSuite/RNAfold.cgi, accessed on 22 October 2024) was used to predict the secondary structure and analyzed the structure of mRNA with modified genes. To further improve translation efficiency, we optimized the base preferences in the 5′UTR and 3′UTR regions, as well as within 30 bp downstream of the ATG start codon. This process excluded rare codons and common enzyme cleavage sites, while taking into account the effects of ribosome-binding sites, microRNA, and the base preference at the 5′ end of the genes on gene regulation. The final optimized sequence is shown in Figure 1F, with a total length of 1328 bp. The red part represents the codon-optimized sequence of *Fad3*, and the black parts are the 3′UTR and 5′UTR. The ECOR1 and BamH1 enzyme cleavage sites and the protective bases (shown in blue) were added at both ends.

### 3.2. Vector Construction of the Fad3 Gene

**Figure 2:**
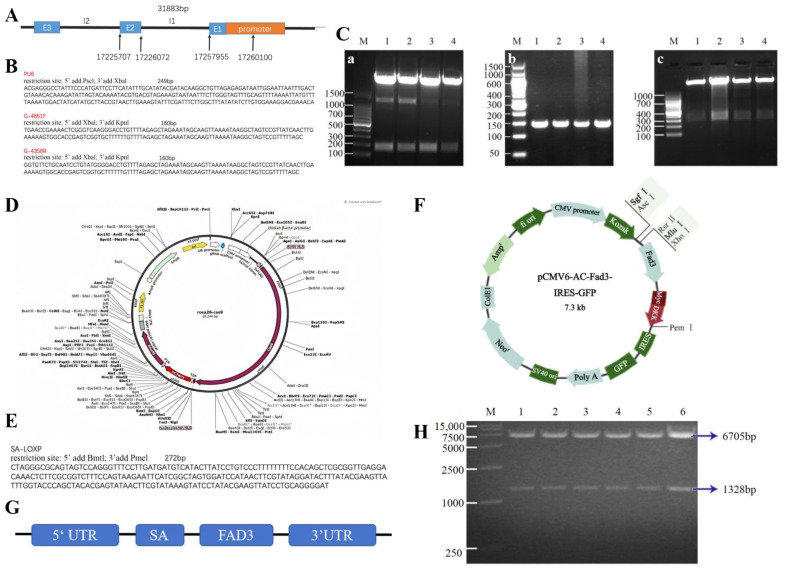
Vector construction of the *Fad3*. (**A**) The structure of the bovine Rosa26 locus. (**B**) U6 promoter and sgRNA sequence. (**C**) Identification of the link between the U6 promoter and sgRNA, where a is the restriction enzyme identification of the U6 promoter in the intermediate vector, with M: 100 bp marker; b is the restriction enzyme identification of the sgRNA in the intermediate vector, with M: 50 bp marker; c is the restriction enzyme identification of the U6 sgRNA-Cas9 vector, with M: DL1000 marker; and 1, 2, 3 and 4 correspond to the 4347 sgRNA intermediate vector, the 4358 sgRNA intermediate vector, the 4844 sgRNA intermediate vector, and the 4851 sgRNA intermediate vector, respectively. (**D**) A schematic diagram of the U6 sgRNA-Cas9 vector. (**E**) SA sequence information. (**F**) A schematic diagram of the Fad3 vector. (**G**) A schematic diagram of the Fad3 vector sequence. (**H**) The electrophoresis diagram of the double-enzyme digestion process of the Fad3-GFP vector, with M: DL15,000 marker, where 1, 2, 4, 5, 7, and 8 correspond to different monoclonal plasmids.

The bovine Rosa26 is 31,883 bp in length and consists of three exons and two introns (Figure 2A). The fragment of intron 1 near exon 2 was selected as the target region, and the site with fewer restriction enzyme sites was chosen to insert the foreign gene. Based on the sequence information, four suitable sgRNAs were designed (Table 1).

The selected Cas9 vector incorporates the red fluorescent gene mCherry, enabling the transfection status to be determined based on the expression of red fluorescent protein. The U6 promoter, which drives sgRNA expression, was inserted into the Cas9 vector. Both the U6 promoter and the sgRNA sequence were synthesized, with their sequence information presented in Figure 2B. First, the U6 promoter was linked to sgRNA, and the U6 sequence was linked to the intermediate vectors of sgRNA 4851, 4358, 4844, and 4347, utilizing the restriction enzyme sites PscI and XbaI. The successful ligation of these vectors was confirmed through restriction enzyme digestion (Figure 2C). Next, the U6 sgRNA fragment was excised from the intermediate vector using PscI and KpnI and linked to the Cas9 vector (Figure 2C). This process produced the final U6 sgRNA-Cas9 vector (Figure 2D).

CRISPR-Cas9 technology was employed to target and cleave the intron of the Rosa26 gene in bovine cells. Simultaneously, a donor vector containing the Fad3 gene expression cassette flanked by homologous arms was designed for homology-directed repair (HDR). To enhance the expression levels of the Fad3 gene, an intron splicing sequence (SA sequence) was integrated into the expression cassette (Figure 2E). Additionally, BrmtI and PmeI restriction endonuclease recognition sites were incorporated at both ends of the cassette to facilitate cloning and verification. A 3′-homologous arm (1400 bp) and the 5′-homologous arm (1600 bp) were obtained from the genome of Luxi cattle with restriction endonuclease sites, which are Agel + Bst17I and Spe1 + Nhe1, respectively. This process built the 5′arm-SA-Fad3-3′arm vector (Figure 2F,G). The successfully constructed donor vector plasmid was digested with Bst17I and XbaI, the large fragments were recovered (Figure 2H), and the cells were co-transfected with the Cas9 vectors.

### 3.3. Fad3 Vector Transfection and Screening of Positive Cells

**Figure 3:**
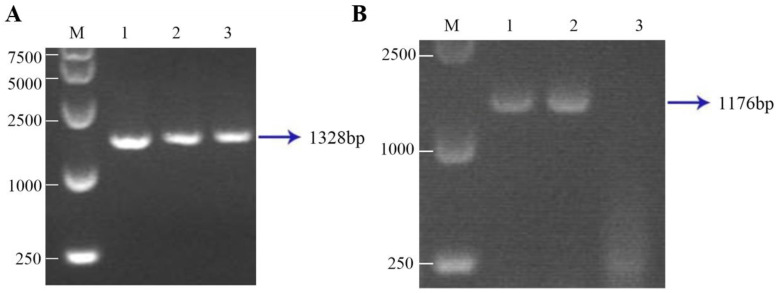
Transfection of the Fad3 vector and the screening of positive cells. (**A**) A diagram of PCR electrophoresis of the *Fad3* gene in transgenic cells. M: DL15,000 marker; 1: Fad3 plasmid; 2 and 3: DNA of transgenic cells. (**B**) RT-PCR electrophoresis diagram of transgenic cells. M: DL15,000 marker; 1 and 2: cDNA of transgenic cells; 3: H_2_O.

The 5′arm-SA-Fad3-3′arm gene expression box was transfected into the fetal fibroblast cells of Luxi yellow cattle by using a liposome transfection method. After the single cells grew into clones, these continued to subculture, and a portion of cells was harvested for *Fad3* gene identification. Genomic DNA was extracted from these cells and then specific PCR was performed. The positive rate was 30.2%. This indicated that the foreign gene *Fad3* had been successfully integrated into the genome of the transgenic cells (Figure 3A). RT-PCR electrophoresis showed that the mRNA expression of *Fad3* could be detected in the transgenic cells (Figure 3B). The primers used for RT-PCR identification of Fad3-positive single-cell clones are shown in Table 2.

The positive transgenic cells were collected for fatty acid analysis. The results, as presented in Table 3, demonstrated that the knock-in of the *Fad3* gene led to a change in the fatty acid content of the cells. Specifically, the ratio of n-6 unsaturated fatty acids to n-3 unsaturated fatty acids decreased from 2.2 to approximately 1.5, representing a reduction of 31.8%. This implied that the codon-optimized Fad3 gene can be correctly expressed in mammalian cells and exert its biological functions.

### 3.4. Generation of Fad3 Transgenic Cattle

**Figure 4:**
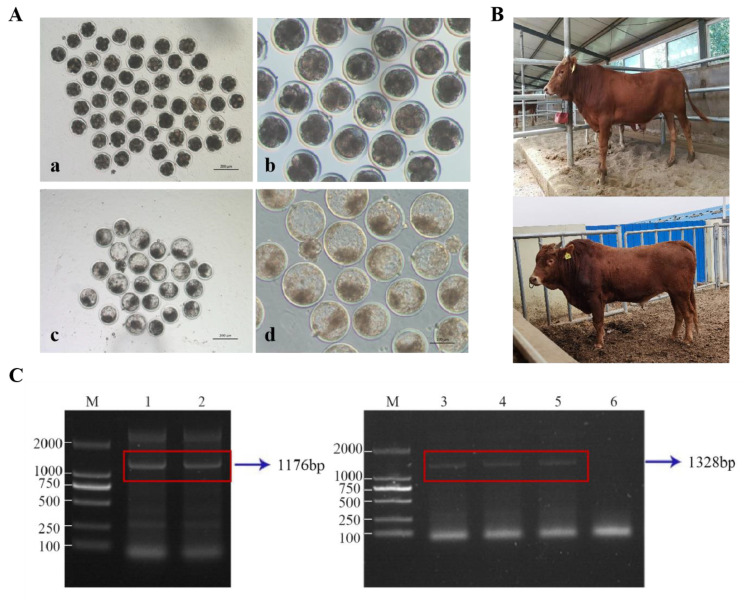
Preparation of *Fad3* transgenic cattle. (**A**) Cloned embryos with *Fad3* gene transferred. (**a**,**b**) Eight-cell stage embryos; (**c**,**d**) blastocysts. (**B**) Adult *Fad3* transgenic cloned cattle. (**C**) Identification of *Fad3* transgenic cloned cattle, with M: DL2000.

To generate Fad3 transgenic cloned embryos, morphologically normal *Fad3* transgenic cells were selected as nuclear donors and subsequently transferred into enucleated oocytes. The reconstructed embryos were fused, activated, and cultured to blastocyst stage. A total of 202 *Fad3* transgenic cloned blastocysts were obtained (Table 4, Figure 4A). Of these, 106 well-formed blastocysts were selected and transferred into the uteri of 53 recipient cows synchronized for estrus in five batches. Sixty days after embryo transfer, rectal pregnancy examinations revealed that 10 recipient cattle were pregnant. At 120 days, seven cows were still pregnant. Eventually, five cows came to term and gave birth to five calves, one of which survived. These results are summarized in Table 5 and Figure 4B. Analysis of ear tissue samples from the cloned offspring by polymerase chain reaction (PCR) and reverse transcription polymerase chain reaction (RT-PCR) confirmed that the calves were *Fad3*-positive (Figure 4C and Supplementary Material S2).

Fatty acid analysis of *Fad3* transgenic cloned bovine cells showed that the fatty acid content had changed. The ratio of n-6 unsaturated fatty acids to n-3 unsaturated fatty acids decreased from 3484 to 2.78 (*p* < 0.01). This suggests that the codon-optimized *Fad3* gene promotes the conversion of n-6 unsaturated fatty acids to n-3 unsaturated fatty acids in mammalian cells (Table 6). Therefore, transgenic cattle with the integrated *Fad3* gene have been successfully obtained.

## 4. Discussion

Outside of *Caenorhabditis elegans* [27], there are no reported instances of animals, including humans, possessing desaturase genes that can alter the ratio of n-6 to n-3 fatty acids. Mammals are obligatory consumers of these essential fatty acids, relying on their diets to supply the necessary n-6 and n-3 polyunsaturated fatty acids (PUFAs). To enhance the nutritional value of dietary sources with health-promoting PUFAs, many researchers are now exploring the development of genetically modified animals that carry fatty acid desaturase genes. Flax, an important oilseed crop in China, stands out due to its high content of α-linolenic acid, linoleic acid, oleic acid, palmitic acid, and stearic acid, among other unsaturated fatty acids [28]. Fad3 is a member of the Δ15 fatty acid dehydrogenase family that converts linoleic acid to α-linolenic acid in plants.

In previous studies, researchers had made significant progress using the *Fat-1* gene of C. elegans in transgenic cells and animal models. In rat cardiomyocytes heterologously expressing the *Fat-1* gene, various n-6 PUFAs were converted to their corresponding n-3 PUFAs, reducing the n-6/n-3 PUFA ratio, making it a valid animal model for subsequent studies [29,30,31,32,33,34]. This approach has also been applied to domestic animals, successfully producing *Fat-1* transgenic pigs [12,13,35,36], cattle [23,37,38], and sheep [22,39,40,41], in which the exogenous transgene function and polyunsaturated fatty acids were highly expressed in fat, muscle, and milk. Comprehensive biosafety assessments of these transgenic animals have shown that integration of the *Fat-1* gene does not cause changes in the host’s genome, appearance, physiological structure, metabolic activities, health, and reproductive abilities. Moreover, the progeny of these genetically modified animals exhibit normal development with no significant differences when compared to their non-transgenic counterparts [42]. In our previous studies, we carried out beneficial experiments on *Fad3* transgenic cells and animal models. After the flaxseed-derived *Fad3* gene was transfected into mouse C2C12 cells, the content of n-6 PUFAs was significantly decreased and the content of n-3 PUFAs was significantly increased. In *Fad3* transgenic mice, we observed that the expression trend of *Fad3* gene varied significantly across different tissues and organs at both the mRNA and protein levels. Specifically, the highest levels of *Fad3* mRNA were detected in the liver, while lower levels were found in skeletal muscle, fat, brain, and heart. At the level of fatty acids, the n-6/n-3 ratio was notably reduced in skeletal muscle, brain, and liver tissues, but changes were not observed in fat, ovarian, or testis tissues [43]. The elevated levels of n-3 PUFAs mediated through the *Fad3* gene influenced several key metabolic pathways. Notably, these fatty acids regulated the AKT and PPAR signaling pathways, affecting glycolipid metabolism in skeletal muscle. This regulation led to a significant decrease in the oxidative phosphorylation capacity by inhibiting the activity and gene expression of critical rate-limiting enzymes in the mitochondrial tricarboxylic acid cycle and electron respiratory chain. Consequently, cellular ATP content was reduced, highlighting the profound metabolic changes induced by the *Fad3* gene [44].

The *Fad3* transgenic mouse model may have advantages over *Fat-1* mice in terms of gene function specificity and animal reproductive health. Unlike mammals, the Fad2 enzyme in plant cells can dehydrogenate the 12th C-C single bond of unsaturated fatty acids to a C=C double bond, resulting in n-6 unsaturated fatty acids and n-3 unsaturated fatty acids via the 15th fatty acid desaturase gene. Mammalian expression vectors of Fat-1 and Fad2 genes were used to generate Fat-1/Fad2 transgenic mice which had significantly increased levels of n-3 and n-6 PUFAs in their livers and shared the same fatty acid metabolic pathways as higher plants and microorganisms [45]. The combination of *Fad2* and *Fad3* genes, when transferred together into mice, results in the generation of *Fad2/Fad3* double-transgenic mice. These mice successfully establish their own polyunsaturated fatty acid biosynthesis pathway, with the *Fad2* enzyme responsible for the de novo synthesis of n-6 PUFAs and the *Fad3* enzyme further converting these n-6 PUFAs to n-3 PUFAs. Compared to *Fad3* mice, the levels of both n-3 and n-6 PUFAs are significantly increased in the *Fad2/Fad3* double-transgenic mice. This unique capability allows the *Fad2-Fad3* double-transgenic mice to spontaneously transform n-9 monounsaturated fatty acids (MUFAs) into n-6 PUFAs and then into n-3 PUFAs, thus achieving de novo synthesis of n-3 type PUFAs [24].

In this study, *Fad3* transgenic cattle were successfully obtained by transfecting cells with the Fad3 vector followed by somatic cell nuclear transfer. With the improvement in the theoretical basis of somatic cell reprogramming, the techniques and methods are also improving. At present, the most commonly used methods include transcription factor-induced induced pluripotent stem cells, cell fusion, cytoplasmic incubation, and somatic cell nuclear transfer techniques [46]. Compared to other technologies, only somatic cell nuclear transfer technology can eventually re-induce most of the differentiated adult cells to develop into complete animal individuals [47]. But the cloning efficiency of somatic cells is still relatively low [48]. There were developmental abnormalities in SCNT embryos before and after implantation and in the perinatal period. Even if the fetus is born normally, there will be defects in the digestive, cardiovascular, reproductive, and skeletal systems after birth, which will eventually lead to the death of the body. The main cause for low cloning efficiency and abnormal embryo development is incorrect or incomplete epigenetic reprogramming of the donor nucleus [49,50]. Currently, the most commonly used methods to improve the reprogramming state of donor cells are drug therapy or regulation of the expression of key genes associated with embryonic development [51,52,53,54], but the improvement in the birth rate of cloned animals remains limited. Therefore, using multi-omics analysis to elucidate the reprogramming factors and mechanisms involved in SCNT may be an effective way to improve our understanding of embryonic development and the developmental ability of cloned embryos. In future research, it is still necessary to improve the development efficiency of SCNT embryos. With the exposure of various obstacles in the development of cloned embryos, the complexity of cloning research is increasing day by day. Combined with the elimination of multiple barriers, complete reprogramming was achieved, greatly improving cloning efficiency [55].

After being codon-optimized, the *Fad3* gene suitable for the mammalian genome was obtained, and an expression vector without a resistance gene and a marker gene was constructed. After cell transfection and screening, we obtained *Fad3* transgenic cells. Detection and identification of the *Fad3* gene at the cell level indicated that the *Fad3* gene had normal replication, transcription, and translation activities. The desaturation ability of the gene was fully verified, and the fatty acid composition showed the expected changes. The exogenous *Fad3* gene in cattle showed excellent activity, and the composition of fatty acids changed fundamentally, which was completely consistent with the biological function of the *Fad3* gene. *Fad3* transgenic cattle were successfully generated in this study, but the interaction between the exogenous *Fad3* gene and the endogenous genes of transgenic cattle, as well as more detailed biological mechanisms, still need to be further explored.

## 5. Conclusions

In conclusion, the successful creation of *Fad3* transgenic cattle using CRISPR-Cas9 technology and somatic cell nuclear transfer (SCNT) represents a significant breakthrough in agricultural biotechnology. This study demonstrates the effectiveness of inserting a codon-optimized *Fad3* gene sequence into bovine fibroblast cells, resulting in cattle with a notably enhanced content of n-3 PUFAs. The gas chromatographic analysis revealed a significant increase in n-3 PUFA levels and a reduction in the n-6-to-n-3 PUFA ratio, indicating a shift toward a more desirable fatty acid profile in the meat of these transgenic animals. The implications of this research could extend beyond agriculture, fostering further exploration into the health benefits of n-3 PUFAs and the potential for genetic interventions in animal husbandry to create healthier food sources. Overall, *Fad3* transgenic cattle not only have an enhanced quality of beef but also pave the way for innovative approaches in nutrition and health studies.

## Figures and Tables

**Table 1:** sgRNA sequence design.

Site	Sequence	PAM Sequence	GC%
4844–4863 (F)	ACCACGATGAACCGAAAACT	CGG	52%
4851–4870 (F)	TGAACCGAAAACTCGGGTCA	AGG	52%
4347–4366 (F)	GAGCTGAACCACAAGACGTT	AGG	52%
4358–4377 (RC)	GGGTATGTCCTAACGTCTTG	TGG	52%

**Table 2:** Primers for PCR identification of *Fad3*-positive single-cell clones.

Gene Name	5′-3′ Forward Primers	5′-3′ Reverse Primers
Fad3	AATGTGGAGAAGGATGAGTC	ATGTCGTGGTGGATGTTG

**Table 3:** The analysis of PUFAs in fibroblasts of the Control group (*n* = 3) and Fad3 transgenic positive fibroblasts (*n* = 3).

Items	Cell		*p* Values
PUFAs	Control (*n* = 3)	Positive (*n* = 3)	
C18: 2n-6	1.40 ± 0.29	2.11 ± 0.09 *	0.0306
C18: 3n-6	0.05 ± 0.05	1.05 ± 0.08 ***	0.0001
C20: 4n-6	5.37 ± 0.31	3.48 ± 0.12 **	0.0014
n-6 total	7.22 ± 0.15	6.42 ± 0.19 *	0.0102
C18: 3n-3	0.52 ± 0.07	0.85 ± 0.10 *	0.0190
C20: 5n-3	1.75 ± 0.22	2.05 ± 0.16 ^ns^	0.1928
C22: 6n-3	1.18 ± 0.16	1.63 ± 0.09 *	0.0233
n-3 total	3.257 ± 0.22	4.55 ± 0.27 *	0.0065
n-6/n-3	2.26 ± 0.17	1.47 ± 0.09 **	0.0043

Note: Positive specifically refers to Fad3-positive transgenic cells that have passed the screening. *: *p* < 0.05; **: *p* < 0.01; ***: *p* < 0.001; ns: not significant.

**Table 4:** Preparation of *Fad3* transgenic cattle embryos.

No.	No. of Cultured Oocytes	No. of Mature Oocytes (%)	No. of Nuclear Transfer Oocytes	No. of Fused Embryos (%)	No. of Activated Embryos	No. of Cleavage Embryos (%)	No. of Blastocysts (%)
1	305	242 (79.3)	215	191 (88.9)	180	142 (78.9)	48 (26.7)
2	361	260 (72.0)	244	208 (85.2)	196	170 (86.7)	53 (27.0)
3	372	261 (70.2)	237	195 (82.3)	185	155 (83.8)	55 (29.7)
4	353	257 (72.8)	231	192 (83.5)	184	139 (75.5)	46 (25.0)
Total	1391	1020 (73.3)	927	786 (84.8)	745	606 (81.3)	202 (27.1)

**Table 5:** Pregnancy and birth rates of *Fad3* transgenic cattle.

No.	No. Blastocysts	No. Recipient Cattle	Pregnancy at 60 Days (%)	Pregnancy at 120 Days (%)	Pregnancy at 210 Days (%)	Birth (%)	Survival (%)
1	26	13	3 (23.1)	2 (15.4)	2 (15.4)	2 (15.4)	0
2	30	15	1 (6.7)	0	0	0	0
3	28	14	4 (28.6)	3 (21.4)	1 (7.1)	1 (7.1)	1 (7.1)
4	22	11	2 (18.2)	2 (18.2)	2 (9.5)	2 (9.5)	0
Total	106	53	10 (18.8)	7 (13.2)	5 (9.4)	5 (9.4)	1 (1.9)

**Table 6:** Analysis of PUFAs in ear tip fibroblasts from calves born by artificial insemination (Control, *n* = 3) and *Fad3* transgenic calves (*n* = 3).

Items	Cattle		*p* Value
PUFAs	Control (*n* = 5)	Fad3 (*n* = 5)	
C18: 2n-6t	32.34 ± 3.93	71.262 ± 2.57 ***	0.0000
C18: 2n-6c	3.502 ± 0.43	6.02 ± 1.66 *	0.0350
C18: 3n-6	1.304 ± 0.38	9.146 ± 2.20 ***	0.0005
C20: 4n-6	5.94 ± 0.32	68.284 ± 1.91 ***	0.0000
n-6 total	44.292 ± 2.97	154.712 ± 5.53 ***	0.0000
C20: 3n-3	3.62 ± 0.42	34.096 ± 1.90 ***	0.0000
C20: 5n-3	5.22 ± 0.32	7.198 ± 1.10 ***	0.0128
C22: 6n-3	5.36 ± 0.73	15.638 ± 2.27 ***	0.0001
n-3 total	15.016 ± 1.37	55.932 ± 4.53 ***	0.0000
n-6/n-3	3.484 ± 0.46	2.78 ± 0.14 *	0.0427

Note: * *p* < 0.05 and *** *p* < 0.001.

## Data Availability

All of the data generated or analyzed during the present study are available from the corresponding author upon reasonable request.

## Supplementary Materials

The following supporting information can be downloaded at https://www.mdpi.com/article/10.3390/ani15010093/s1: Material S1: Humanization of the *Fad3* gene; Material S2: The sequencing results of the *Fad3* gene.
