# Supplementary material for: Generation of Codon-Optimized Fad3 Gene Transgenic Bovine That Produce More n-3 Polyunsaturated Fatty Acids
Source: Animals (Basel). 2025 Jan 3;15(1):93. doi: 10.3390/ani15010093 (PMC11718938; doi:10.3390/ani15010093)
Supplement: Supplementary file 1 [file animals-15-00093-s001.zip › animals-3283422-supplementary.pdf]

Supplementary Material S1. Humanization of the Fad3 gene.

Step 1: Based on the data provided on the NCBI website, select the Linum usitatissimum Fad3b gene (LuFad3B): accession number (ACCESSION): DQ116425, CDS: 1176 bp, encoding amino acids (391 aa).

Step 2: Based on the Codon Usage Database, the codon preference characteristics of the relevant DNA sequences are counted in the CDS regions of all known genes of the two species cattle (Bos taurus) and human (Homo sapiens). Considering that humans are the ultimate beneficiary, codon optimisation for DQ116425 is performed according to the human codon preference comparison table.

All codons of the LuFad3B gene are fully optimised to the optimal codons of Homo sapiens (this standard is the current software optimisation principle). Of the 1176 bases, a total of 238 bases need to be changed. The base changes are mainly that A and T are transformed into C and G. After optimisation, the GC content increases from 50.22% to 65.22%.

Step 3: Given that species exhibit a greater propensity for the utilisation of specific codons, the abundance of particular codon transfer RNA (tRNA) species and the scarcity of rare codon tRNAs within cells, the gene obtained in Step 2 is subjected to a secondary modification in accordance with the maximum abundance of human tRNAs. The rare codons in the exogenous gene are converted to synonymous codons that occur frequently in the recipient cell, and some of the optimal codons are replaced with suboptimal codons.

Step 4: In light of the impact of DNA methylation on gene expression, it is remodelled again according to the CG-enriched region in the upstream regulatory region of the gene, while at the same time, synonymous codon substitution is carried out in the region with a relatively high degree of CG enrichment within the gene.

Step 5: Considering the stability of the exogenous gene mRNA and the influence of the mRNA secondary structure on the translation efficiency, when optimizing, it is necessary to avoid the formation of special mRNA secondary structures that hinder expression. Through the RNA Structure software to predict and analyze the secondary structure of the modified gene mRNA, optimize the structure that is not conducive to mRNA expression. The secondary structure of the optimized sequence is shown in Figure S1.

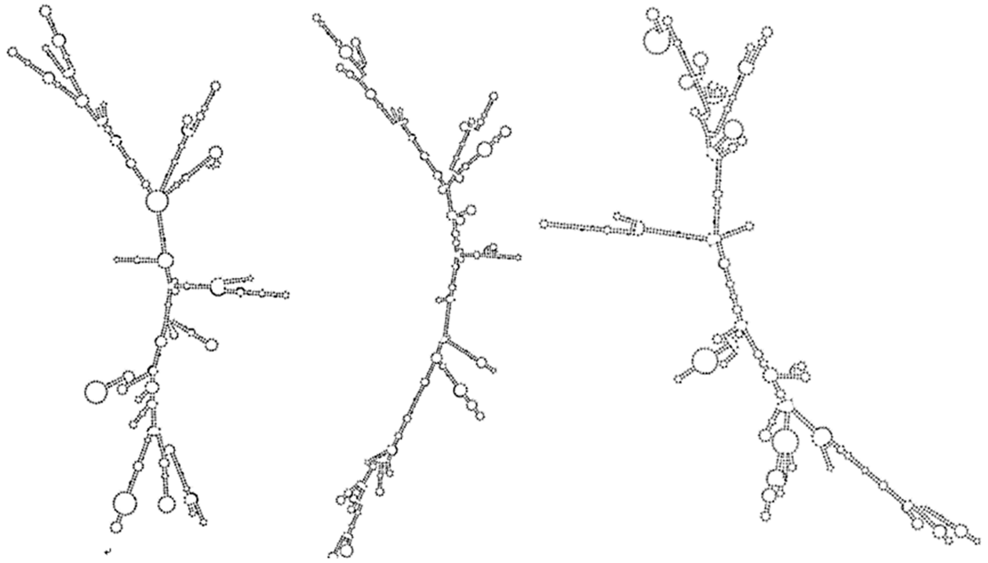

Figure S1. The secondary structure after optimizing the Fad3 gene.

Step 6: Considering the effectiveness of the ribosome binding site and the influence of the base preference at the 5' end of the gene on the efficiency of gene translation initiation, by comparing the base preference near the start codon ATG and its upstream of the housekeeping gene, the base preference in the 5'UTR region of the modified HuFad3B gene and within 30 bp downstream of ATG is optimized.

Step 7: Considering the inhibitory effect of genomic DNA methylation on the efficiency of gene expression, partially exclude the CG dinucleotides in the 5'UTR region downstream of the gene transcription start site and inside the gene optimized in Step 5, and optimize the base AT preference. The specific sequence is as follows:

>LuFad3B Total:1328 5′UTR: 30bp
ttcaaaactgtggctctgcaggaccaaact
>ACTB Total:1852 5′UTR: 84bp
accgccgagaccgcgtccgccccgcgagcacagagcctcgcctttgccgatccgccgcccgtccacacccgccgccagctcacc
>GAPDH Total:1401 5′UTR: 174bp
ggctgggactggctgagcctggcgggaggcggggtccgagtcaccgcctgccgccgcgcccccggtttctataaattgagccc
gcagcctcccgcttcgctctctgctcctcctgttcgacagtcagccgcatcttcttttgcgtcgccagccgagccacatcgctcagac
acc
>EEF1A1 Total:3528 5′UTR: 63bp
ctttttcgcaacgggtttgccgccagaacacaggtgtcgtgaaaactacccctaaaagccaaa
>TPT1 Total:829 5′UTR: 93bp
ccccccgagcgccgctccggctgcaccgcgctcgctccgagtttcaggctcgtgctaagctagcgccgtcgtcgtctcccttcagtc
gccatc

Step 8: Considering the influence of microRNA on gene regulation, optimize the 3'UTR region of the HuFad3B gene after the transformation in Step 6, and replace it with reference to the housekeeping gene:

>LuFad3B Total:1328 3′UTR: 123bp
Tgatatttggctctgatatatgcaggctgtttatcttgtcctttgttcgtttctttctcccagaaacaaattctctgtttctatgtttctctgt
ctctcccgcccccagctttctttctgag
>ACTB Total:1852 3′UTR: 640bp
Gcggactatgacttagttgcgttacaccctttcttgacaaaacctaacttgcgcagaaaacaagatgagattggcatggctttattt
gtttttttgttttgttttggttttttttttttttggcttgactcaggatttaaaaactggaacggtgaaggtgacagcagtcggttgga
gcgagcatcccccaaagttcacaatgtggccgaggactttgattgcacattgttgttttttaatagtcattccaaatatgagatgcgt
tgttacaggaagtcccttgccatcctaaaagccaccccacttctctctaaggagaatggcccagtcctctcccaagtccacacaggg
gaggtgatagcattgctttcgtgtaaattatgtaatgcaaaatttttttaatcttcgccttaatactttttttattttgttttattttgaatgat
gagccttcgtgccccccccttcccccttttttgtcccccaacttgagatgtatgaaggcttttggtctccctgggagtgggtggaggca
gccagggcttacctgtacactgacttgagaccagttgaataaaagtgcacaccttaaaaatgaaaaaaaaaaaaaaaaaaaaaa
aaaaaaaaaaaaaaaaaaa
>GAPDH Total:1401 3′UTR: 219bp
Gacccctggaccaccagccccagcaagagcacaagaggaagagagagaccctcactgctggggagtccctgccacactcagt
cccccaccacactgaatctcccctcctcacagttgccatgtagacccctTgaagaggggaggggcctagggagccgcaccttgtc
atgtaccatcaataaagtaccctgtgctcaaccaaaaaaaaaaaaaaaaaaaa
>EEF1A1 Total:3528 3′UTR: 2076bp
atattatccctaatacctgccaccccactcttaatcagtggtggaagaacggtctcagaactgtttgtttcaattggccatttaagttta

gtagtaaaagactggttaatgataacaatgcatcgtaaaaccttcagaaggaaaggagaatgttttgtggaccactttggttttctt
ttttgcgtgtggcagttttaagttattagtttttaaaatcagtactttttaatggaaacaacttgaccaaaaatttgtcacagaattttga
gacccattaaaaaagttaaatgagaaacctgtgtgttcctttggtcaacaccgagacatttaggtgaaagacatctaattctggtttt
acgaatctggaaacttcttgaaaatgtaattcttgagttaacacttctgggtggagaatagggttgttttccccccacataattggaa
ggggaaggaatatcatttaaagctatgggagggttgctttgattacaacactggagagaaatgcagcatgttgctgattgcctgt
cactaaaacaggccaaaaactgagtccttgtgttgcatagaaagcttcatgttgctaaaccaatgttaagtgaatctttggaaacaa
aatgtttccaaattactgggatgtgcatgttgaaacgtgggttaaaatgactgggcagtgaaagttgactatttgccatgacataa
gaaataagtgtagtggctagtgtacaccctatgagtggaagggtccattttgaagtcagtggagtaagctttatgccagtttgatg
gtttcacaagttctattgagtgctattcagaataggaacaaggttctaatagaaaaagatggcaatttgaagtagctataaaattag
actaatctacattgcttttctcctgcagagtctaatacctttatgctttgataattagcagtttgtctacttggtcactaggaatgaaact
acatggtaataggcttaacaggtgtaatagcccacttactcctgaatctttaagcatttgtgcatttgaaaaatgcttttcgcgatcttc
ctgctgggattacaggcatgagccactgtgcctgacctcccatatgtaaaagtgtctaaaggtttttttttggttataaaaggaaaat
ttttgcttaagtttgaaggataggtaaaattaaaggacatgctttctgtttgtgtgatggtttttaaaaatttttttaagatggagttctt
gttgcccaggctagaatgcaatggcaaaatctcactgcaatctcctcctcctgggttcaagcaattctcctacttcagcctcccaagt
agctgggattacaggcatgtgctaatttggtgtttttaatagagatgaggtttttccatgttggtcaggctggtctcaaactcctgac
cttaggtgatcgcctcggcctcctaaagtgctggaattacaggcatgagccaccatgcctggccaggacatgtgttcttaaggaca
tgctaagcaggagttaaagcagcccaagagataaggcctcttaaagtgactggcaatgtgtattgctcaagattcaaaggtactt
gaattggccatagacaagtctgtaatgaagtgttatcgttttccctcatctgagtctgaattagataaaatgccttcccatcagccagt
gctctgaggtatcaagtctaaattgaactagagatttttgtccttagtttctttgctatctaatgtttacacaagtaaatagtctaagatt
tgctggatgacagaaaaaacaggtaaggcctttaatagatggccaatagatgccctgataatgaaagttgacacctgtaagattt
accagtagagaattcttgacatgcaaggaagcaagatttaactgaaaaattgttcccactggaagcaggaatgagtcagtttactt
gcatatactgagattgagattaacttcctgtgaaacccagtgtcttagacaactgtggcttgagcaccacctgctggtattcattaca
aacttgctcactacaataaatgaattttaagctttaaaaaaaaaaaaaaaaaa

>TPT1 Total:829 3’UTR: 217bp

caaatgtggcaattattttggatctatcacctgtcatcataactggcttctgcttgtcatccacacaacaccaggacttaagacaaatg
ggactgatgtcatcttgagctcttcatttattttgactgtgatttatttggagtggaggcattgtttttaagaaaaacatgtcatgtagg
ttgtctaaaaataaaatgcatttaaactcatttgagag

Step 9: By comparing the composition of the human genome triplet and the exclusion of rare codons, and also excluding common enzyme cleavage sites, splicing sites, and translation termination sites, finally the following optimized sequence is obtained:

```
               10        20        30        40        50        60        70        80        90       100
DQ1164 ATGAGCCCTCCAAACTCAATGAGTCCCACCACCAACGGCAATGGTGTGGCTATGAATGGGGCGAAGAAGCAGCTCGATTTCGACCCGAGTGCTGCCCCCC
       :::  :::::: ::::: :::  :::::::::::::::::: :::::::::::::::::::: ::::::::::: ::::: :::::    ::::::::::
DQ1164 ATGTCCCCTCCCAACTCCATGTCCCCCACCACCAACGGCAACGGTGTGGCTATGAATGGGGCCAAGAAGCAGCTGGATTTTGACCCCTCCGCTGCCCCCC
               10        20        30        40        50        60        70        80        90       100
              110       120       130       140       150       160       170       180       190       200
DQ1164 CTTTCAAGATTGCAGACATCCGTGCTGCAATTCCGCCGCATTGCTGGGTGAAGAACCCCTGGAGGTCGCTCAGCTACGTCCTGAGAGACCTCCTTGTCAT
       : ::::::::::: :::::::: ::::: :: :: :: :: ::::::::::::::::::::: : :: ::   :::::::::::::::::: :: :::::
DQ1164 CCTTCAAGATTGCCGACATCCGCGCTGCCATCCCCCCCCACTGCTGGGTGAAGAACCCCTGGCGCTCCCTGTCCTACGTCCTGAGAGACCTGCTGGTCAT
              110       120       130       140       150       160       170       180       190       200
              210       220       230       240       250       260       270       280       290       300
DQ1164 CCTCAGCTTCGCCGTTGCGGCGGCAAAGCTGGACAGCTGGACTTTCTGGCCTCTTTACTGGGTTGCTCAAGGAACCATGTTCTGGGCAGTCTTTGTTCTT
       :::   ::::::::: ::::: :: :::::::::  ::::::::::::::: :: :::::::: ::::: :: :::::::::::::: :::::::: ::
DQ1164 CCTGTCCTTCGCCGTGGCGGCTGCCAAGCTGGACTCCTGGACTTTCTGGCCCCTGTACTGGGTGGCTCAGGGCACCATGTTCTGGGCCGTCTTTGTGCTG
              210       220       230       240       250       260       270       280       290       300
              310       320       330       340       350       360       370       380       390       400
DQ1164 GGACATGATTGTGGCCATGGGAGCTTCTCAGACATCTGGTTGTTGAACAATGTGATGGGACATATACTCCATTCCTCAATCCTCGTACCTTACCATGGAT
       :: :: :: :::::::::::   :::::: ::::::::: :: ::::::: :::::::: :: :: :: :: ::::: ::::: :: :: :::::::: :
DQ1164 GGCCACGACTGTGGCCATGGCTCCTTCTCCGACATCTGGCTGCTGAACAACGTGATGGGCCACATCCTGCACTCCTCCATCCTGGTGCCCTACCATGGCT
              310       320       330       340       350       360       370       380       390       400
              410       420       430       440       450       460       470       480       490       500
DQ1164 GGAGAATTAGCCACAAGACCCATCACCAGAATCACGGCAATGTGGAGAAAGATGAATCCTGGGTTCCTCTACCGGAGAAAGTGTACAAGAGCTTGGATAC
       :::::::   :::::::::::: :::::::: ::::::::::::::::: ::::: :::::::: ::::: :: ::::: :::::::::  : :::: ::
DQ1164 GGAGAATCTCCCACAAGACCCACCACCAGAACCACGGCAATGTGGAGAAGGATGAGTCCTGGGTGCCTCTGCCCGAGAAGGTGTACAAGTCCCTGGACAC
              410       420       430       440       450       460       470       480       490       500
              510       520       530       540       550       560       570       580       590       600
DQ1164 CAGCACTAAGTTCATGAGGTTCACCATTCCTCTCCCAATGTTTGCTTATCCTATCTACTTGTGGACGAGAAGTCCGGGGAAGAAAGGGTCGCATTTCAAC
       :  ::::::::::::: : :::::::: :::::::: ::::::::::::::::::::: :::::::  :    :: :: ::::: :: :: :: ::::::
DQ1164 CTCCACTAAGTTCATGCGCTTCACCATCCCTCTCCCCATGTTTGCTTATCCTATCTACCTGTGGACCCGTTCCCCCGGCAAGAAGGGCTCCCACTTCAAC
              510       520       530       540       550       560       570       580       590       600

              610       620       630       640       650       660       670       680       690       700
DQ1164 CCATACAGCGACCTATTCGCACCAAACGAGAGGGCAGCGGTCTTGATTTCAACATTGTGCTGGACAGCCATGGCCTTACTCCTCTGCTACTCATCGTTCA
       :: :::  :::::: ::::: :: :::::: : ::::::::: :::: :: ::  :::::::::: ::::::::: : ::::: :::::::: :: ::::
DQ1164 CCCTACTCCGACCTGTTCGCCCCCAACGAGCGCGCAGCGGTCCTGATCTCCACCCTGTGCTGGACCGCCATGGCCCTGCTCCTGTGCTACTCCTCCTTCA
              610       620       630       640       650       660       670       680       690       700
              710       720       730       740       750       760       770       780       790       800
DQ1164 TATACGGCTTCGCTCCGGTCCTCAAAATCTACGGCGTACCTTATCTGATATTCGTGGCATGGCTCGACATGGTGACCTACCTTCATCACCACGGGTACGA
       : :::::::::::::: ::::: :: ::::::::::: ::::::::::: :::::::: ::::: ::::::::::::::::: :: :::::::: :::::
DQ1164 TCTACGGCTTCGCTCCCGTCCTGAAGATCTACGGCGTGCCTTATCTGATCTTCGTGGCCTGGCTGGACATGGTGACCTACCTGCACCACCACGGCTACGA
              710       720       730       740       750       760       770       780       790       800
              810       820       830       840       850       860       870       880       890       900
DQ1164 GCAGAAGCTGCCGTGGTACAGAGGCAAAGAATGGAGCTACCTACGTGGAGGGCTGACGACCGTTGATCGAGATTACGGGGTCATCAACAACATCCACCAT
       :::::::::::: ::::::::::::::::: :::  :::::: :: :: :: ::::: ::::: :: :: :::::::: ::::::::::::::::::::
DQ1164 GCAGAAGCTGCCCTGGTACAGAGGCAAAGAGTGGTCCTACCTGCGCGGCGGCCTGACCACCGTGGACCGCGATTACGGCGTCATCAACAACATCCACCAC
              810       820       830       840       850       860       870       880       890       900
              910       920       930       940       950       960       970       980       990      1000
DQ1164 GACATTGGCACCCATGTCATTCACCATCTCTTCCCTCAAATGCCACACTATCACCTTGTGGAAGCGACTCAGGCAGCGAAGCACGTGCTGGGGAAGTACT
       :::::::::::::: ::::: ::::: ::::::::::: ::::: ::::::::::: ::::: :: ::::::::::: :::::::::::::: :::::::
DQ1164 GACATTGGCACCCACGTCATCCACCACCTCTTCCCTCAGATGCCCCACTATCACCTGGTGGAGGCCACTCAGGCAGCCAAGCACGTGCTGGGCAAGTACT
              910       920       930       940       950       960       970       980       990      1000
             1010      1020      1030      1040      1050      1060      1070      1080      1090      1100
DQ1164 ACAGAGAGCCGAAGAAATCAGGGCCTTTCCCATTCCACTTGTTTGGGTACTTGGTAAGGAGCCTGGGCGAGGATCACTACGTTAGCGACACAGGCGACGT
       :::::::::: :::::::: ::::::::::: :::::: ::::::: ::: ::::  :   ::::::::::::::::::::: :::::::: ::::::::
DQ1164 ACAGAGAGCCCAAGAAATCCGGGCCTTTCCCCTTCCACCTGTTTGGCTACCTGGTGCGCTCCCTGGGCGAGGATCACTACGTGAGCGACACCGGCGACGT
             1010      1020      1030      1040      1050      1060      1070      1080      1090      1100
             1110      1120      1130      1140      1150      1160      1170
DQ1164 CGTTTTCTATCAGTCTGACCCACATATTCCCAAGTTCCGTACCAGCAGTGCCACCACCAAGTCCAAATCCAGCTGA
       ::: ::::::::::::::::: :: :::::::::::::: ::::::   ::::::::::::::::: :::::::::
DQ1164 CGTGTTCTATCAGTCTGACCCCCACATTCCCAAGTTCCGCACCAGCTCCGCCACCACCAAGTCCAAGTCCAGCTGA
             1110      1120      1130      1140      1150      1160      1170
```

Supplementary Material S2. The sequencing results of the Fad3 gene

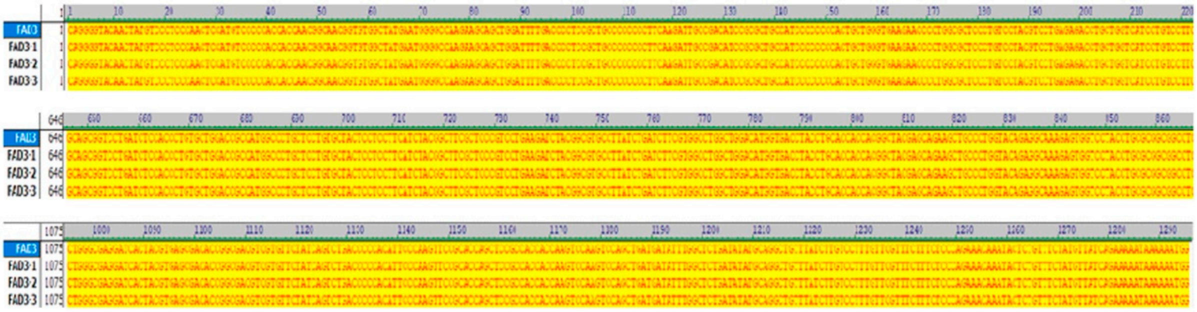

The appendix is an optional section that can contain details and data supplemental to the main text—for example, explanations of experimental details that would disrupt the flow of the main text but nonetheless remain crucial to understanding and reproducing the research shown; figures of replicates for experiments of which representative data is shown in the main text can be added here if brief, or as Supplementary data. Mathematical proofs of results not central to the paper can be added as an appendix.
